# Predicting Tumor Mutational Burden From Lung Adenocarcinoma Histopathological Images Using Deep Learning

**DOI:** 10.3389/fonc.2022.927426

**Published:** 2022-06-08

**Authors:** Yi Niu, Lixia Wang, Xiaojie Zhang, Yu Han, Chunjie Yang, Henan Bai, Kaimei Huang, Changjing Ren, Geng Tian, Shengjie Yin, Yan Zhao, Ying Wang, Xiaoli Shi, Minghui Zhang

**Affiliations:** ^1^ Department of Oncology, Municipal Hospital of Chifeng, Chifeng, China; ^2^ Geneis Co., Ltd., Beijing, China; ^3^ Qingdao Geneis Institute of Big Data Mining and Precision Medicine, Qingdao, China; ^4^ Department of Oncology, Inner Mongolia Medical University, Hohhot, China

**Keywords:** tumor mutation burden, lung cancer, digital pathology, immunotherapy, deep learning, difference analysis

## Abstract

Tumor mutation burden (TMB) is an important biomarker for tumor immunotherapy. It plays an important role in the clinical treatment process, but the gold standard measurement of TMB is based on whole exome sequencing (WES). WES cannot be done in most hospitals due to its high cost, long turnaround times and operational complexity. To seek out a better method to evaluate TMB, we divided the patients with lung adenocarcinoma (LUAD) in TCGA into two groups according to the TMB value, then analyzed the differences of clinical characteristics and gene expression between the two groups. We further explored the possibility of using histopathological images to predict TMB status, and developed a deep learning model to predict TMB based on histopathological images of LUAD. In the 5-fold cross-validation, the area under the receiver operating characteristic (ROC) curve (AUC) of the model was 0.64. This study showed that it is possible to use deep learning to predict genomic features from histopathological images, though the prediction accuracy was relatively low. The study opens up a new way to explore the relationship between genes and phenotypes.

## Introduction

At present, the most advanced treatment for non-small cell lung cancer is the combination of immunotherapy and chemotherapy. Among them, PD-L1 expression is a common biomarker in immunotherapy response, but a large number of patients with low PD-L1 expression and some patients with h7igh PD-L1 expression are suitable for this treatment plan (1–3). Therefore, searching for new predictors of immunotherapy response is crucial.

TMB is an important biomarker for predicting immunotherapy (4–6). TMB is a measure of the total number of non-synonymous somatic mutations per megabase in the coding region of the tumor genome (7). Tumors with high TMB are thought to express a variety of neoantigens. A number of studies have shown that the response of patients with advanced solid tumors to immunotherapy is related to high TMB (4, 7, 8). Therefore, researchers will pay more attention to the existence of TMB when implementing immunotherapy. Therefore, there is an urgent need for a low-cost, fast and reliable TMB detection method.

Whole-exome sequencing is the gold standard for measuring TMB. However, due to technical limitations and high costs, whole-exome sequencing has not been promoted in the field of clinical oncology (9). Therefore, clinicians usually use low-cost next-generation targeted gene sequencing. When the gene-directed therapy guided by the target panel is used and tested, the turnaround time is usually about three weeks. At present, clinicians are trying to re-adjust the purpose of targeted sequencing analysis to facilitate the prediction of TMB when using whole-exome sequencing, and use the default technical method to normalize the number of mutant genes found in the sequencing area (10). Therefore, to obtain a robust normalized TMB, paired normal samples and a larger panel size (at least about 2 megabases) are required (11). The increase in panel size is directly proportional to the cost, which forces a trade-off between the depth of sequencing and the number of patients for each sequencing run. In most clinical treatment processes, the turnaround time of targeted sequencing analysis usually exceeds the prescribed time due to the limitations of various problems. Therefore, it is very beneficial to develop an alternative and convenient method to assess TMB.

Although early histopathologists have recognized the connection between individual genetic mutations and certain cancer morphological phenotypes, they did not consider the application of TMB. Previous machine learning used manual features in histopathological images (12, 13) to distinguish subtypes (14) and predict recurrence (15, 16) and survival outcomes (17). With the rise of deep learning, it is expected to achieve more robust and accurate predictions in biomedical images (18). Deep convolutional networks have shown good results in tumor detection (19–21) and distinguishing subtypes of non-small cell lung cancer (22, 23) and other cancers (24, 25).

Here, we used the ResNet18 deep learning model and used the formalin-fixed paraffin-embedded (FFPE) hematoxylin and eosin (H&E) stained lung adenocarcinoma (LUAD) histopathology images from the Cancer Genome Atlas (TCGA, https://www.cancer.gov/tcga) to predict TMB status. We tried to develop a deep learning method for LUAD, because compared with other cancers of TCGA, we can obtain a large number of sequence and image data of patients with non-small cell lung cancer, and used TMB as a biomarker for tumor treatment. ResNet18 is a convolutional neural network (CNN) that achieves advanced performance on ImageNet. We convinced that with the further development of deep learning and clinical verification, deep learning can provide a potential alternative detection method to determine TMB, while reducing the diagnosis cycle and cost consumption. Our results showed that we could use deep learning techniques to detect previously unexplored features in histopathological images that have been clinically proven to be useful.

## Methods

### Data Processing

**Figure 1:**
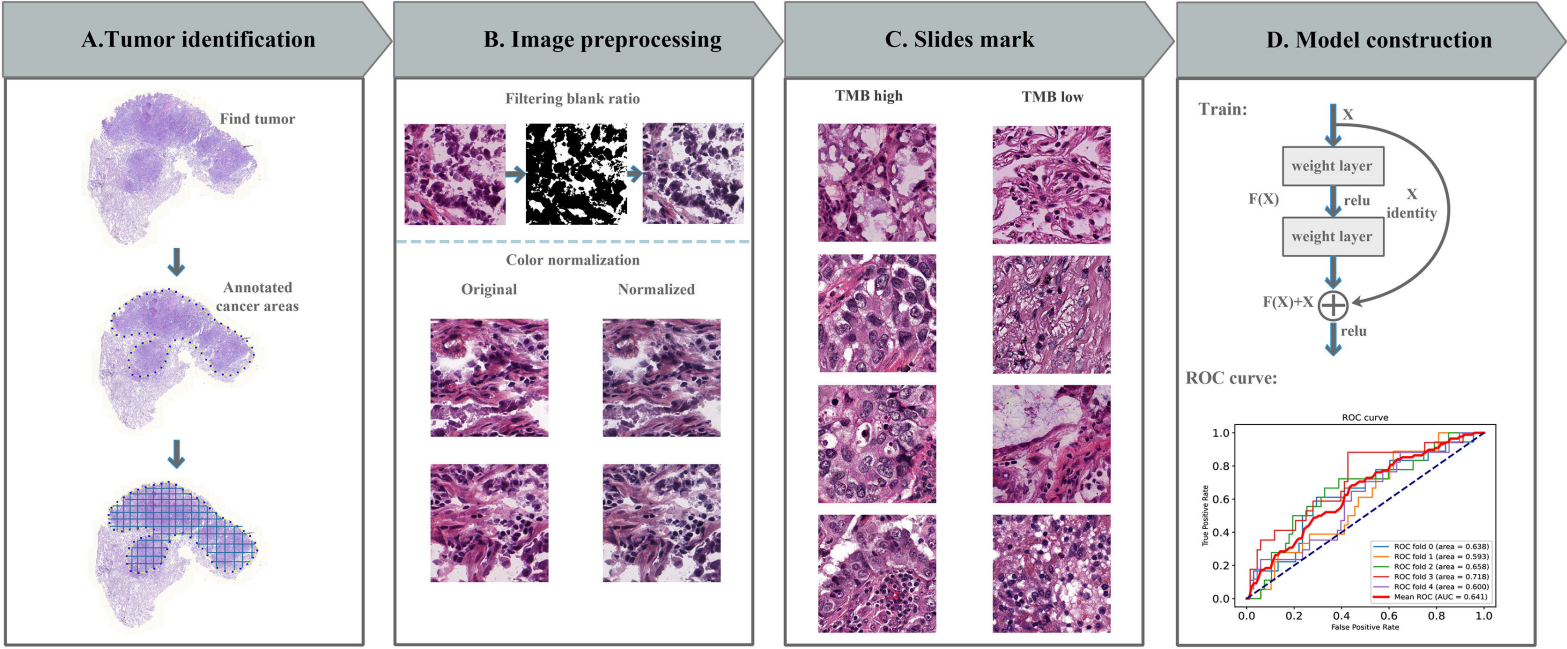
Computational pipeline for predicting TMB in lung cancer. **(A)** The tumor area was annotated by a professional pathologist and cut into 512*512 image blocks. **(B)** Image processing includes noise reduction (discarding image blocks with a blank rate greater than 30%) and color normalization (Macenko method). **(C)** Divide TMB high and low with 10 as the threshold. **(D)** The prediction model was constructed with residual network, the model was tested with 5-fold cross-validation, and receiver operating characteristic (ROC) curve was used to evaluate the model.

In this study, we used whole-slide images (WSIs) of LUAD from TCGA (https://portal.gdc.cancer.gov/repository/). In addition, the clinical information and next generation sequencing results of these patients were downloaded and analyzed. All LUAD WSIs were stored in SVS format and adjusted to 0.5 um per pixel at the same magnification (40x). These WSIs were then marked with tumor areas by a professional pathologist (**Figure 1A**). We set a TMB threshold (10, corresponding to a maftools count of 400) to mark each patient’s TMB status.

Considering that the image pixels of WSIs were too large, it cannot be directly used as the input of the deep learning network. Therefore, we divided WSIs into 512*512 pixels slides, each WSI can be divided into tens of thousands image slides. And then deleted slides with less information (e.g. blank rate over 30%) (15). The error of the manual production process and the difference between stains and scanners will produce a color difference between digital sections, which will cause errors in the subsequent analysis work. Therefore, we performed color normalization using the Macenko method in the Tia toolbox software package (**Figure 1B**) (26).

In the pipeline, we promised each patient and its related slides were allocated to a training or testing dataset to ensure that there was no overlap problem and to ensure the accuracy of the final test results (**Figure 1C**). The final model predicted the TMB status according to the features of each patient, so as to assist the doctor in giving the corresponding immunity treatment plan.

### Deep Learning on Histopathological Images

We used a deep neural network based on the ResNet18 architecture to predict the probability of TMB status for each tile (**Figure 1D**). ResNet18 builds a 18-layer deep convolutional neural network by repeatedly using two residual blocks, Conv Block and Identity Block. The last of the ResNet18 architecture is a fully connected layer for soft-max operations. The output produced two normalized probability predictions. We choose a TMB value of ten as the segmentation threshold. The model parameters were initialized using pre-trained weights form the ImageNet competition (27). Using backpropagation to train all parameters of the model during model training. The loss function was defined as the cross entropy between the true label and the predicted probability.

### Patient-Level Prediction

As shown in **Figure 1**, the TMB status of each image block can be predicted by our prediction model, and these predicted image blocks were divided into two categories: TMB high and TMB low by the soft-max function. The image patches of each patient were then aggregated together to predict the probability of high or low TMB for each patient. Specifically, we divided the number of patches predicted to be high TMB per patient by the total number of patches per patient. If its probability was greater than 0.5, the LUAD cancer patient was predicted to have high TMB, otherwise, the patient was defined as low TMB. And the patient’s TMB status is the standard for subsequent provision of corresponding treatment plans.

### Performance Evaluation

After the model was trained, cross-validation was used for model testing and performance evaluation. We used the percentage of correctly classified slices to aggregate the probability of each slide. We used the scikit-learn in the python library to calculate the ROC curve and the corresponding AUC value in the case of prediction.

In addition, we performed survival analysis of true and predicted TMB status of these 427 lung adenocarcinoma patients. Overall survival time was calculated from the date of surgery to the date of death or last follow-up contact. Survival curves were estimated using the Kaplan-Meier multiplicative limit method (E. L. 28). Differences in predicted survival outcomes between high and low TMB groups were compared by log-rank test.

### Hyperparameter and Model Selection

Model selection and all hyperparameters were based on the performance of the validation dataset, including the use of the ResNet18 model, optimization of parameters, and so on. The validation dataset is only used after the model is developed and used to introduce all the results obtained in this research. In the process of model evaluation, the methods and models we use are not modified in any way to ensure independence assumptions between datasets. To avoid the effects of the algorithm falling into local optimal solutions and data noise, we employed the SGD + momentum optimizer, where momentum assigns a value of 0.9. For every seven epochs, the learning rate of the parameters decayed by 0.1 times. Moreover, 30 epochs were trained throughout the process.

### Statistical Analysis

All statistical analysis was conducted using R software. We used statistical methods to analyze the differences of clinical characteristics in 427 cases of TCGA lung adenocarcinoma patients with high and low TMB data.

In clinical, for continuous clinical characteristic variables such as age and the number of cigarettes per day, we used the Wilcoxon rank sum test method to analyze (29). For non-continuous variables such as tumor stage, we used the fisher’s exact test method to analyze. P-values less than 0.05 were considered statistically significant.

DESeq2 method of R package was used to analyze the difference of mRNA in TMB high and low groups. DEseq2 requires the input data to be an unnormalized matrix of integers (30). At the same time, we also performed GO (gene ontology) enrichment and KEGG (Kyoto Encyclopedia of Genes and Genomes) pathway enrichment analysis on mRNA, and annotated from three aspects: BP (biological process), MF (molecular function), and CC (cellular component). The GO enrichment and KEGG calculation method and formula are the same, and they are calculated using the hypergeometric test:


$$P=1-\sum_{i=0}^{m}\frac{\left(\begin{array}{c} M\\ i \end{array}\right)\left(\begin{array}{c} N-M\\ n-i \end{array}\right)}{\left(\begin{array}{c} N\\ n \end{array}\right)}$$

Among them, ‘N’ is the number of genes with Pathway annotation in all genes. ‘n’ is the number of differentially expressed genes in N. ‘M’ is the number of genes annotated as a specific pathway in all genes. ‘m’ is the number of differentially expressed genes annotated as a specific Pathway. The calculated P value is further corrected for multiple testing to obtain the corrected p-value (that is, the Q value). Usually we take Q value ≤ 0.05 as a threshold, and a pathway that satisfies this condition is defined as a pathway that is significantly enriched in differentially expressed genes (31).

## Results

### Smoking and Age Were Significantly Correlated With TMB Status

In this study, 468 patients’ sequencing results were downloaded from TCGA and 427 H&E-stained WSIs of formalin-fixed paraffin-embedded tumor tissue sections of all available LUAD cases were obtained. Therefore, after matching the patient information, 427 patients were finally included in this study. The clinical information of these 427 patients was also obtained from TCGA and performed statistical analysis, and some of the clinical information was shown in **Table 1**. According to the TMB threshold, the number of TMB high was 88, and the number of TMB low was 339 among 427 patients with LUAD.

**Table 1:** Summary of the general clinical information of patients with lung adenocarcinoma.

Clinicopathologic variable	Category	TCGA LUAD
Sample type	H&E stained sections	427
Age	<=50 >50 NA	33 379 15
Cigarettes_per day	<2 >=2 NA	140 157 130
Tumor stage	I II III IV NA	238 102 55 25 7

**Figure 2:**
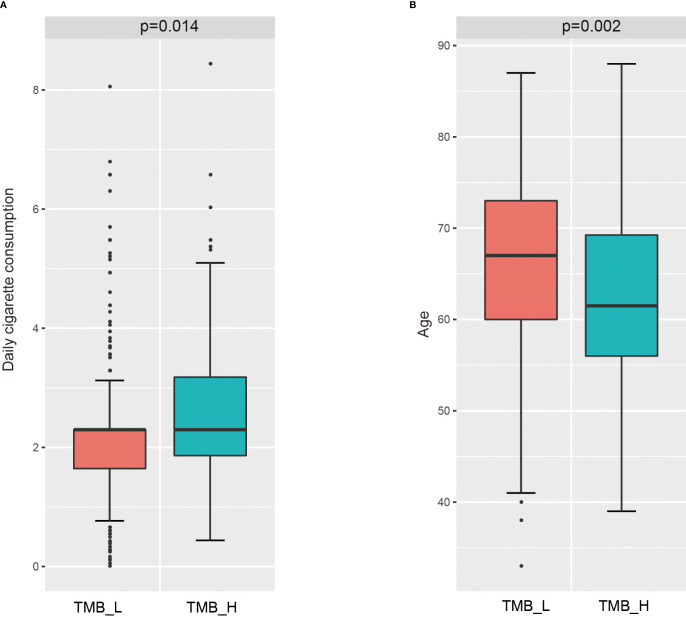
Correlation of several clinical features with TMB status. **(A)** Correlation analysis between daily cigarette consumption and TMB status. **(B)** Relationship between ages and TMB status.

Then, we analyzed the relationship between these clinical characteristics and TMB status, the results were shown in **Figure 2**. Daily cigarette consumption was significant different (p=0.014) in patients between TMB-H group and TMB-L group (**Figure 2A**). The daily smoking volume of TMB-H group was significantly higher than that of TMB-L group. Besides, the age of patients in TMB-H group was significantly lower than that in TMB-L group (**Figure 2B**). Whereas, there was no significant correlation between tumor stage and TMB status, with a P value of 0.09, which was shown in **Supplementary Figure 1**.

### Many Differential Genes Were Identified Between TMB-H and TMB-L Samples

**Figure 3:**
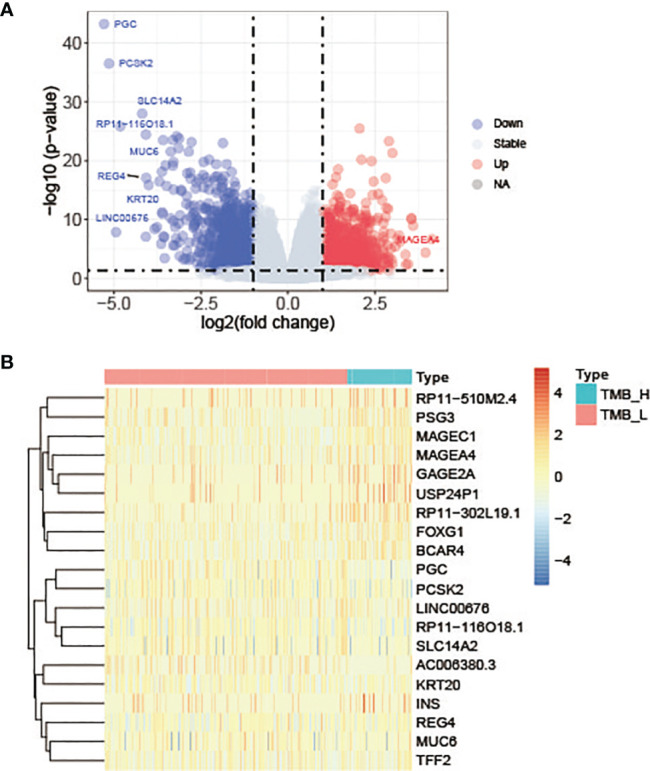
Differentially expressed genes in TMB-H group compared with TMB-L group. **(A)** Volcano plots of the differentially expressed genes. Red dots represent up-regulated genes, blue dots represent down-regulated genes and gray dots represent genes with no significant expression differences. **(B)** Heatmap of top 10 up-regulated genes and down-regulated genes in TMB-H group compared to TMB-L group.

The mRNA sequencing data of cancer tissue samples from 427 patients with lung adenocarcinoma were downloaded from TCGA. And then these patients were divided into TMB-H group and TMB-L group according to the value of TMB. To explore whether the changes of high or low expression of some key genes will directly lead to the increase of the total number of gene mutations, that is, affect TMB status, the gene expression differences between the two groups were analyzed using DESeq2. With log2 |fold change| ≧ 1 and p value ≤ 0.05 as the threshold, we got 2140 significantly differentially expressed genes in TMB-H group compared with TMB-L group. Among these genes, 960 genes were up-regulated and 1180 genes were down-regulated in TMB-H group (**Figure 3A**). The top 10 up-regulated genes and down-regulated genes in TMB-H group compared to TMB-L group were shown respectively in **Figure 3B**.

### The Differential Genes Between TMB-H and TMB-L Are Significantly Enriched in Many GO Terms and KEGG Pathways

**Figure 4:**
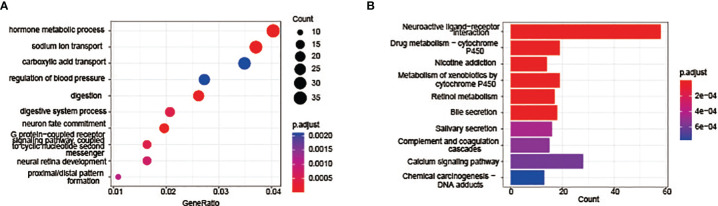
Functional enrichment and pathway analysis results of differentially expressed genes. **(A)** Top 10 enriched GO terms annotated in biological process. **(B)** Top 10 KEGG pathways of the differentially expressed genes.

Go enrichment analysis and KEGG pathway enrichment analysis were further carried out to clarify the functions and signaling pathways involved in these differential expressed genes. GO enrichment analyses include three aspects: BP, CC and MF. The top ten Go terms in the biological process were displayed in **Figure 4A**, including hormone metabolic process, sodium ion transport, carboxylic acid transport, regulation of blood pressure, digestion, digestive system process, neuron fate commitment, G protein-coupled receptor signaling pathway etc. KEGG pathway analysis results indicated that differentially expressed genes are mainly enriched in the neuroactive ligand-receptor interaction pathway, Calcium signalling pathway, drug metabolism-cytochrome P450 pathway and nicotine addiction pathway (**Figure 4B**). Genes in these pathways may regulate the mutation and repairment of DNA.

### Histopathological Images Could be Used to Predict TMB

After preprocessing, the histological images of H&E staining in TMB high group were labeled as 1, meaning positive sample, and those in TMB low was labeled as 0. Small tiles of pathological images from the same patient are divided into the same dataset to ensure that information is not leaked. Then the ResNet18 model was employed to train the samples, and 5-fold cross validation was used to split the samples and verify the results.

**Figure 5:**
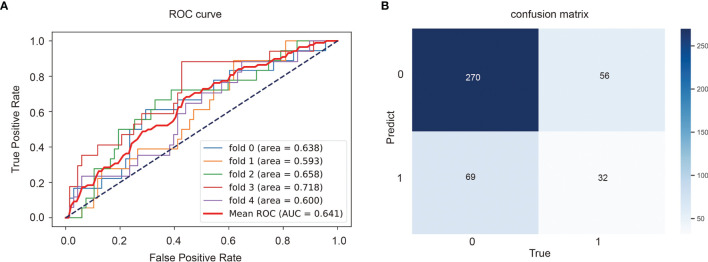
Performance of the TMB predict model based on histopathological images. Area under the receiver operator curves (ROCs) **(A)** and the confusion matrix **(B)** of the deep learning model.

The classification model we developed will be used to predict TMB status of the entire slide image of a given patient, which will provide a certain basis for pathologists’ later diagnosis and treatment. The ROC curve and AUC of the predict model were shown in **Figure 5A** and the confusion matrix was illustrated in **Figure 5B**. The TMB predict model achieved a relatively good performance, with an area under the curve (AUC) of 0.641.

**Figure 6:**
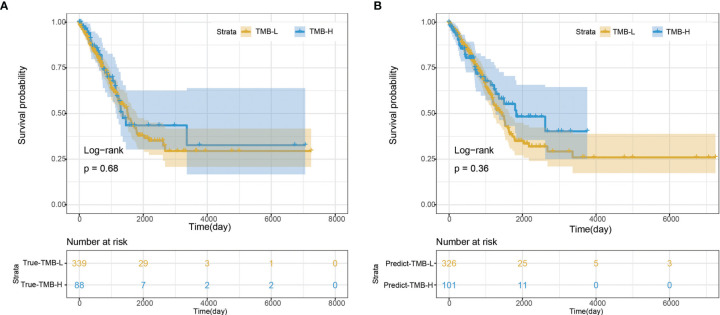
Survival analysis. **(A)** Survival analysis based on the true clinical data. **(B)** Survival analysis based on the predict result of TMB status by H&E-stained histological images.

We further evaluated the performance of the deep learning model by survival analysis (**Figure 6**). As shown in **Figure 6A**, there was no significant difference in the survival time between TMB-H group and TMB-L group from the real clinical statistics. The survival analysis results of the two groups were not significantly different based on the predict TMB status by H&E-stained histological images, which was consistent with the real statistical data (**Figure 6B**).

## Discussion

TMB plays an important role in immunotherapy response, and it is an immunotherapeutic biomarker recommended by National Comprehensive Cancer Network (NCCN) guidelines. In this study, we proved that TMB can be evaluated using digitized FFPE histopathological images in LUAD, though the prediction accuracy was relatively low. This may be related to the small number of training datasets or the threshold division of TMB. In addition, the inconsistent calculation method of TMB may also lead to inaccurate data division, which will have an impact on the results.

At present, the generalization ability of the model is affected by the dataset. In the course of clinical treatment, patients with advanced malignant tumors have relatively small diagnostic biopsy specimens, which may come from many different potential sites, including the liver, lymph nodes and other sites. In order to test the practical application of our method to clinical samples, it is necessary for us to train and test in these different scenarios. In addition, TCGA FFPE images can be highly enriched in tumor cells, but this does not reflect the real tissue samples used in the biopsy process. However, experts can manually mark the tumor area on the slide image and apply ResNet18 to the area of interest. In view of the results obtained using FFPE section samples, it was feasible to use frozen sections for research in the future.

Our deep learning model was only trained to determine whether TMB was above a selected threshold, rather than predicting specific TMB values. And, it would be better if we had an independent validation set to validate our model. As a clinically relevant measurement standard, TMB usually requires additional and laborious testing. We can predict TMB from the H&E-stained histopathology images of the LUAD datasets in TCGA. In the future, we can predict TMB from H&E images of lung squamous cell carcinoma (LUSC). In addition, our method has certain advantages compared with other diagnostic tools. We can predict the TMB value of each area of the image. This method can represent the heterogeneity of TMB itself or the heterogeneity of histological characteristics related to high TMB.

There are a few limitations of this work. First, we used deep learning to link genome-wide features with histopathological images, which helps to study the spatial heterogeneity of tumors and the relationship between cancer phenotypes and genotypes. Therefore, the use of deep learning is a useful method to improve the current large number of ready-made histopathological images, and helps to prioritize and screen patient samples and follow-up treatment. However, the prediction accuracy of model is relatively low. More advanced machine learning models might be able to improve the accuracy like some recent classification models used in other biological problems (32–34). Second, we used 5-fold cross validation to evaluate the model accuracy. It might be better to find some more independent datasets. However, it is infeasible to find a new dataset with both histopathological images and WES. Finally, TMB is used as an indicator for immunotherapy. It might be more direct to predict the outcomes of immunotherapy directly. However, this is listed as our future work provided that we can find some appropriate datasets.

## Data Availability Statement

The original contributions presented in the study are included in the article/**Supplementary Material**. Further inquiries can be directed to the corresponding authors.

## Ethics Statement

Ethical review and approval was not required for the study on human participants in accordance with the local legislation and institutional requirements. Written informed consent for participation was not required for this study in accordance with the national legislation and the institutional requirements.

## Author Contributions

MZ and XS conceived the project; SY and LW implemented the experiments and analyzed the data; CY, YZ, XZ, YW, HB, YN, and YH prepared the data and performed literature search; KH, CR, and XS wrote the manuscript; all authors revised and approved the final manuscript.

## Conflict of Interest

The authors LW, KH, CR, GT, and XS are employed by Geneis Beijing Co. Ltd.

The remaining authors declare that the research was conducted in the absence of any commercial or financial relationships that could be construed as a potential conflict of interest.

## Publisher’s Note

All claims expressed in this article are solely those of the authors and do not necessarily represent those of their affiliated organizations, or those of the publisher, the editors and the reviewers. Any product that may be evaluated in this article, or claim that may be made by its manufacturer, is not guaranteed or endorsed by the publisher.
