# Supplementary figures and images for: Predicting Tumor Mutational Burden From Lung Adenocarcinoma Histopathological Images Using Deep Learning
Source: Front Oncol. 2022 Jun 8;12:927426. doi: 10.3389/fonc.2022.927426 (PMC9213738; doi:10.3389/fonc.2022.927426)

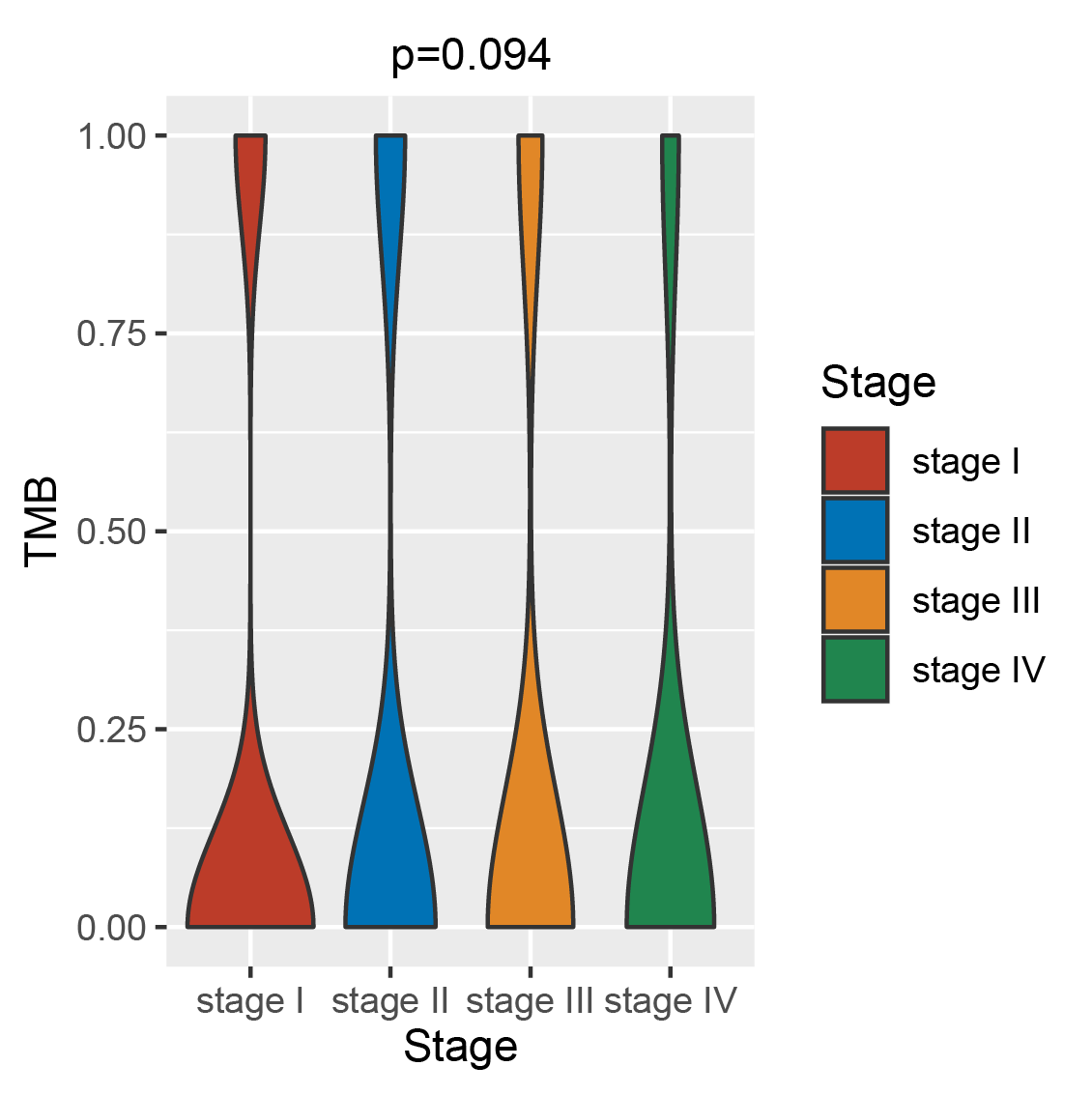

Supplement: Supplementary file 1 [file Image_1.tif]
